# Role of vitamin D and calcium signaling in epidermal wound healing

**DOI:** 10.1007/s40618-022-01893-5

**Published:** 2022-08-13

**Authors:** D. D. Bikle

**Affiliations:** Department of Medicine and Dermatology, University of California San Francisco, San Francisco VA Medical Center, San Francisco, USA

**Keywords:** Vitamin D, Calcium, Keratinocytes, Wounding, p63, Stem cells

## Abstract

**Purpose:**

This review will discuss the role of vitamin D and calcium signaling in the epidermal wound response with particular focus on the stem cells of the epidermis and hair follicle that contribute to the wounding response.

**Methods:**

Selected publications relevant to the mechanisms of wound healing in general and the roles of calcium and vitamin D in wound healing in particular were reviewed.

**Results:**

Following wounding the stem cells of the hair follicle and interfollicular epidermis are activated to proliferate and migrate to the wound where they take on an epidermal fate to re-epithelialize the wound and regenerate the epidermis. The vitamin D and calcium sensing receptors (VDR and CaSR, respectively) are expressed in the stem cells of the hair follicle and epidermis where they play a critical role in enabling the stem cells to respond to wounding. Deletion of *Vdr* and/or *Casr* from these cells delays wound healing. The VDR is regulated by co-regulators such as the Med 1 complex and other transcription factors such as Ctnnb (beta-catenin) and p63. The formation of the Cdh1/Ctnn (E-cadherin/catenin) complex jointly stimulated by vitamin D and calcium plays a critical role in the activation, migration, and re-epithelialization processes.

**Conclusion:**

Vitamin D and calcium signaling are critical for the ability of epidermal and hair follicle stem cells to respond to wounding. Vitamin D deficiency with the accompanying decrease in calcium signaling can result in delayed and/or chronic wounds, a major cause of morbidity, loss of productivity, and medical expense.

## Introduction

Wound healing of the skin involves a complex interaction among a number of cell types including keratinocytes, fibroblasts, endothelial cells, nerves, and inflammatory and immune cells [1]. The process can be thought of as occurring in three phases: the inflammatory phase, the proliferative phase, and the maturation phase. During the inflammatory phase a clot forms to stop the bleeding, and inflammatory cells enter the wound to begin the debridement process, block infection, and secrete cytokines that both advance the inflammatory process and stimulate proliferation and migration of the fibroblasts and keratinocytes. The proliferation and migration of keratinocytes begins the re-epithelialization of the wound while the proliferation and migration of the fibroblasts rebuilds the collagen matrix over which the re-epithelialization occurs. The maturation phase includes the restoration of the epidermis and the collagen cross linking within the matrix that heals the wound.

**Fig. 1:**
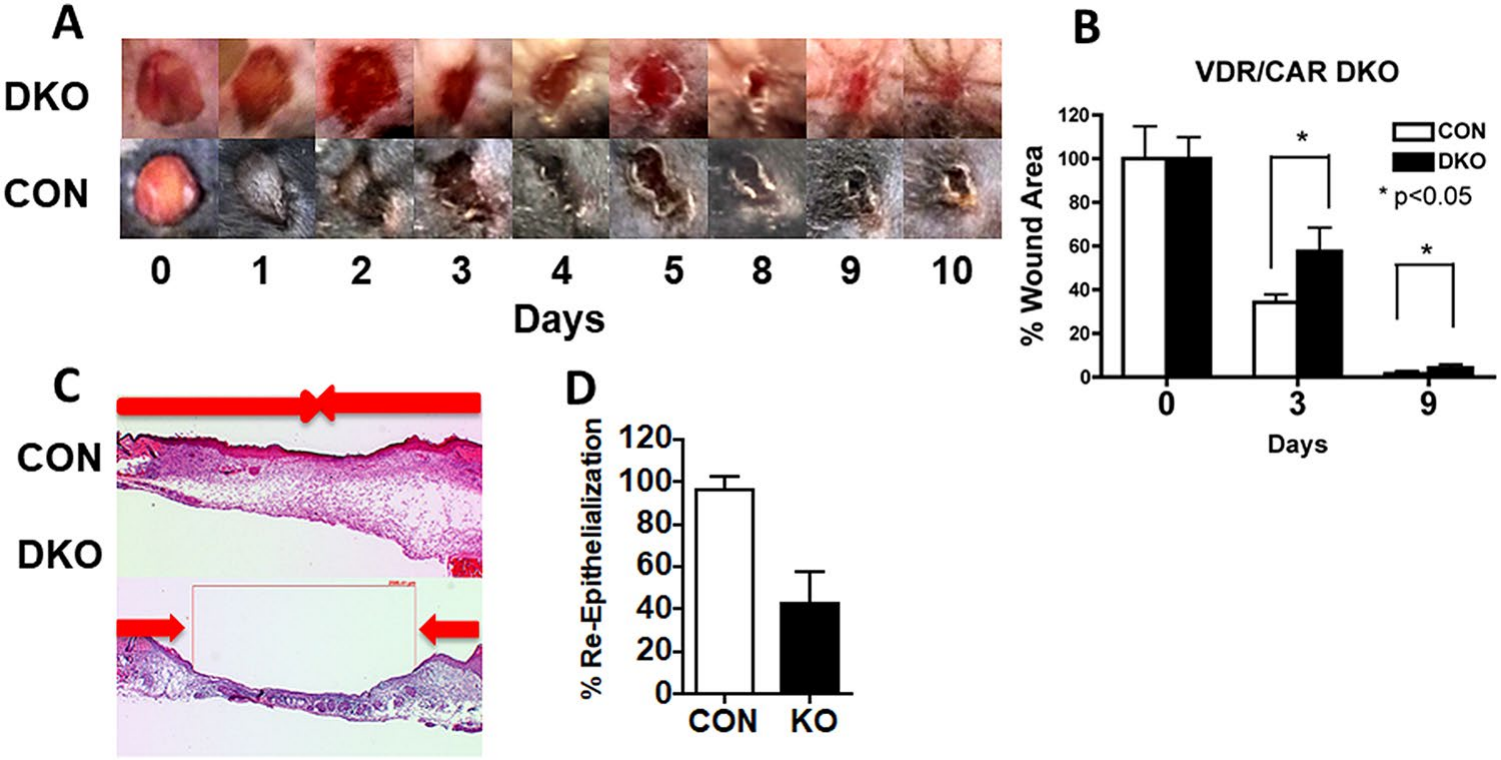
Deletion of the vitamin D and calcium receptors delays wound healing. **A** Six mm full thickness skin biopsies were made on the back of 3 month old DKO mice and their control littermates (CON). The areas of the wounds were measured 0–6 days later and normalized to the original wound area (time 0) in DKO and CON mice. The bars enclose mean ± SD, *p < 0.05 (*n* = 7–8). **B** Representative wound photographs in 0–6 days from DKO and CON mice are shown. **C** Three mm full thickness skin biopsies were made on the back skin of 3 month old DKO and CON, and the re-epithelialization was evaluated histologically at day 3 by H&E staining. Images at higher magnification (boxed) show the edge (white arrows) of epithelial tongues (marked by yellow dotted lines) from which we measured the distance to evaluate the re-epithelialization. **D** Percent re-epithelialization was quantitatively evaluated by analysis of different cross sections (*n* = 6, 3 mice each). Percent re-epithelialization was defined as the distance traveled by both epithelial margins (blue arrows) divided by the distance needed to travel to fully re-epithelialize the wound (red bolts). The bars enclose mean ± SD, statistical significance was evaluated by *t* test *p < 0.05

If this sequence of events does not take place in timely fashion chronic skin wounds are the result. Chronic skin wounds are estimated to affect 6.5 million patients in the US at a cost of over $25 billion [2]. Vitamin D deficiency is associated with an increased risk of chronic wound development. Hu et al., in a study in China of 2925 subjects, found that delayed healing of abdominal surgical wounds correlated inversely with 25OHD levels, with the numbers of patients with delayed healing increasing from 0% in those with 25OHD levels above 30 ng/ml, 13% in those with 25OHD levels above 20 ng/ml, and 36.4% in those with 25OHD levels below 10 ng/ml (personal communication with consent). Like results have been reported by others [3–6]. Vitamin D can impact a number of processes involved with wound healing including the inflammatory and immune response [7] and the proliferation and differentiation of both fibroblasts and keratinocytes [8–10]. Moreover, vitamin D has a major influence on calcium metabolism (and vice versa), and calcium signaling plays a critical role in normal wound healing [11, 12]. We [13] have used a mouse model lacking both the vitamin D receptor (Vdr) and the calcium sensing receptor (Casr) to show this delay in wound healing (Fig. 1).

## Role of stem cells

**Fig. 2:**
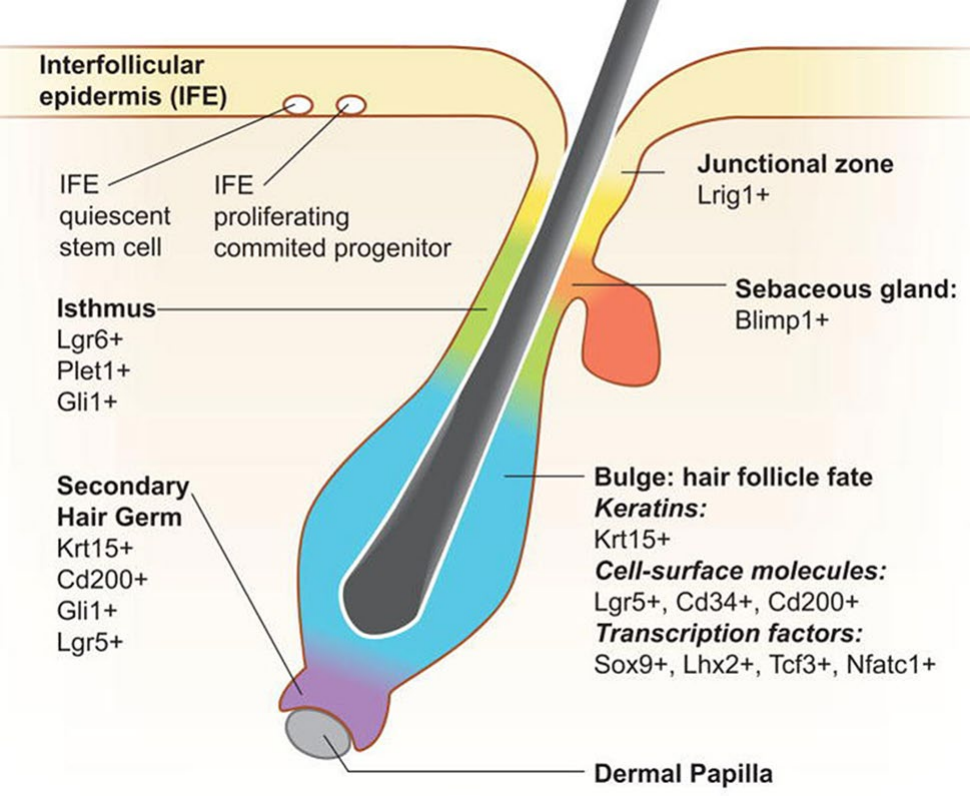
Location of stem cells in the HF and IFE, and the markers used to identify them. Figure adapted from (19)

Our recent studies [13–15] have focused on the roles of vitamin D and calcium signaling in the epidermis in the control of stem cell (SC) activation and function during the initial response to wounding of the skin, a response we propose if defective contributes to poor wound healing. Adult SCs residing in regenerative tissues like the epidermis and hair follicles (HF) play essential roles in the maintenance of those tissues. Understanding the mechanisms controlling adult SC is one of the fundamental goals in the field of skin biology. The skin provides an excellent model system for the study of adult SC in tissue regeneration. Skin epithelia are derived from the ectoderm and differentiate into the interfollicular epidermis (IFE), sebaceous gland (SG) and HF during the embryonic developmental process. After birth, adult SC residing in the basal layer of the epidermis (eSC), junctional zone/ infundibulum (jSC), isthmus (iSC) and bulge (bSC) regions of the HF are responsible for the regeneration of the IFE, SG and the cycling portion of the HF, respectively [16–19]. In the IFE, this regeneration is continuous to produce transient amplifying cells (TAC), which leave the basal layer and differentiate producing proteins such as keratin 1 (Krt1), Krt10, involucrin, filaggrin and loricrin. Cells from the junctional zone/infundibulum in the distal portion of the HF closest to the epidermis contribute to this process. In the isthmus iSC provide cells for the maintenance of the SG. In contrast, the proximal portion of the HF is cyclic, with activation initiated with signals between the bSC and dermal papilla [20–22]. These different SC have distinctive markers in their separate niches (e.g. Lgr5 for bSC, Lgr 6 for iSC, Lrig1 for jSC) but as discussed below these distinctive markers are lost after wounding [23]. Figure 2 is a cartoon showing the location and selected markers of these different stem cell niches.

When the skin is wounded the progeny of SC from all regions of the HF and IFE contribute at least initially [16, 19], although to variable extent. Ito et al. [24] labeled the bSC of the adult mouse using an inducible K15-crePR/R26R transgenic and found that after wounding approximately 25% of the cells in the newly formed epidermis originated from the bulge. However, these cells did not persist. Levy et al. [25] labeled the SC throughout the HF including the infundibulum with a Shh-cre/R26R transgenic and confirmed that SC from other regions of the HF also contribute to re-epithelialization after wounding, and noted that these cells persisted in the regenerated epidermis. We made similar observations with Lrig1-cre expressing SC labeled with Rosa tdTomato in the infundibulum (Oda et al. unpublished data). That said, SC from the HF are not required for re-epithelialization. Langton et al. [26] evaluated wound healing in a mouse model lacking HF, and observed that although healing was delayed, re-epithelialization did eventually occur as does healing in hairless epidermis such as the paw.

Although earlier lineage tracing studies helped define the contributions SC from the different niches made to wound healing, scRNAseq studies have demonstrated a considerably more nuanced picture. Haensel et al. [27] identified 15 clusters (groups of cells with distinguishing transcriptomes) in unwounded skin and 14 clusters after wounding including 4 basal cell subclusters, 3 of which were non proliferating in the epidermis, two spinous subclusters, and four HF subclusters. Of the basal cell subclusters, one was marked by SC genes including Col 17a and ∆Np63, a second with early response gene markers such as fos, jun, and Id1, and a third cluster with growth arrest genes such as Cdkn1a, Irf6, Ovo1, and Sfn. The proliferative subcluster was marked by Ube2c. Krt14 marked all basal subclusters. In the wounded skin the growth arrest cluster type genes were predominant in the leading edge of the re-epithelialization process, the Col17a1 and ∆Np63 were found in the proliferation zone behind the leading edge. Using scPATH analysis the authors concluded that in both wounded and non-wounded skin the epidermal cells follow a path from the Col17a1, ∆Np63 expressing cell through those marked by early response genes to those marked with the growth arrest genes with the transition being faster with wounding. bSC lose the CD34 marker as they migrate into the epidermis during wound healing [23] and enter the same sequence as the eSC. In a similar study Joost et al. [23] examined the time course of wound repair, labeling the Lgr6 and Lgr5 expressing SC (iSC and bSC, respectively) with the fluorescent probe Tomato, and noted the more rapid movement of the Lgr6 + cells into the wound than the Lgr5 + cells, but both expressed an IFE transcriptome as they did so indicating that the response to wounding involved a change in cell fate from producing hair or the sebaceous gland to producing epidermis.

## VDR regulation of SC function

We and others have shown that IFE and HF SC express high levels of VDR [13, 14]. Alopecia is a well described characteristic of mice and humans lacking VDR globally [28–30] due to failure to regenerate the cycling lower portion of the HF after the initial developmental cycle is completed. Cianferotti et al. [31] attributed this to a gradual loss of the proliferative potential in the bSC, which they attribute in part to the loss of VDR. Our results with the epidermal specific VDRKO confirm this and further demonstrate a decrease in eSC numbers [14]. On the other hand Palmer et al. [32] attributed the failure of HF cycling in the *Vdr* null mouse in part to a failure of the progeny of bSC to migrate out of the bulge rather than their loss of proliferative potential suggesting a loss of activation and/or migration of progeny. Our data support this concept as well [14]. Thus our data and those of others indicate that SC loss, decreased activation, and delayed migration of progeny after wounding are present in the VDRKO mouse. These results are consistent with the loss of E-cadherin (Cdh1)/catenin (Ctnn) complex formation in the VDRKO keratinocyte, a complex that requires both calcium and VDR for its formation. The Cdh1/Ctnn complex maintains SC in their niches [33], regulates when SC division is symmetric (to maintain SC numbers) or asymmetric (initiating differentiation) [34], and is essential for the ability of keratinocytes to migrate as a sheet to re-epithelialize the wound [35]. Moreover, as we have shown [36] the Cdh1/Ctnn complex has an important signaling function within the cell that underlies the ability of vitamin D and calcium to regulate keratinocyte proliferation and differentiation and is likely involved with SC activation as discussed below.

## Role of vitamin D and calcium signaling in wound healing

Although VDR is well known as a regulator of epidermal and HF differentiation, its role in wound healing has received considerably less attention. Tian et al. [37] observed that topical 1,25(OH)_2_D enhanced wound healing. Luderer et al. [38] observed that in the global VDRKO, there was a reduction in TGFβ signaling in the dermis, but effects on re-epithelialization were not observed. Our studies have focused on the VDR in keratinocytes. We have observed that re-epithelialization is impaired when the deletion of *Vdr* from keratinocytes is accompanied by either a low calcium diet [14] or a deletion of the *Casr* [13, 39]. CaSR is essential for the keratinocyte response to calcium [40, 41]. Like vitamin D signaling, calcium signaling is expected to play an important role in activating SC to proliferate and their progeny to migrate to re-epithelialize the wound as we and others have demonstrated in the skin [42, 43] and other SC niches [44–46]. We have shown that acute injury of cultured keratinocytes (scratch test) leads to a rapid increase in intracellular calcium (Cai) as does laser damage to whole thickness skin explants [43, 47] or barrier disruption of the epidermis in vivo (a mild form of wounding) [48]. Deletion of *Casr* blunts these rapid changes in Cai after wounding and like deletion of *Vdr* retards wound healing [43].

The superiority of calcium alginate dressings compared to other wound care products [49] indicates the clinical importance of calcium signaling in wound repair. Moreover, the inclusion of vitamin D into such dressings further enhances their effectiveness. Lansdown [50] demonstrated a surge of calcium into the wound within minutes after wounding. Wound fluid was shown to stimulate the migration of keratinocytes, stimulation lost when the fluid was dialyzed, and restored when magnesium and calcium were added back [51]. Formation of the Cdh1/Ctnn complex requires both calcium and vitamin D signaling [36], which as noted above plays an important role in enabling the cell migration required to re-epithelialize the wound [35]. The Cdh1/Ctnn complex, which links to the underlying cytoskeleton via Ctnna1(a-catenin) helps form an epithelial sheet enabling migration of the epidermis across the wound. Calcium stimulation of migration was blocked by inhibitors of PI3K, MAPK, PKCα, and src kinases [52]. The role of PKCα is of particular interest. PKCα is activated by calcium, and plays an important role in calcium induced keratinocyte differentiation [53]. Mice lacking PKCα have delayed re-epithelialization in response to wounding, whereas if the PKCα is constitutively activated, re-epithelialization is accelerated [54]. The authors attribute this regulation of re-epithelialization to a change in the adhesiveness of desmosomes. Lack of PKCα promotes hyperadhesive desmosomes blocking migration, but when PKCα is present and active, the desmosomes become calcium sensitive and less adhesive, permitting migration. These calcium sensitive, less adhesive desmosomes have altered morphology that may contribute to the defect in re-epithelialization in the VDRKO and CaSRKO mice.

Acutely increasing the extracellular calcium concentration (Cao) above 0.1 mM (calcium switch) leads to the rapid redistribution of a number of proteins from the cytosol to the membrane where they participate in the formation of intercellular contacts. These include the CaSR, phospholipase C-γ1 (PLC-γ1) src kinases, and the Cdh1/Ctnn complex with phosphatidyl inositol 3 kinase (PI3K), phosphatidyl inositol 4-phosphate 5-kinase 1α (PIP5K1α), various Ctnns including Ctnna1, Ctnnb1, Ctnnd1 (α and β-catenin, p120). These all play important roles in calcium induced differentiation [40, 55–59], but are also likely to enable the response of the cell to wounding. PI3K and PIP5K1α sequentially phosphorylate PIP and PIP2 to PIP3 that activates PLC-γ1. PLC-γ1 cleaves PIP2 to form IP3 and diacylglycerol; IP3 releases calcium from intracellular stores important for the acute response to wounding, and diacylglycerol along with calcium activates PKCα. As noted above one of the major pathways disrupted in the VDRKO when analyzed by microarray and immunochemistry is the formation of the Cdh1/Ctnn complex. Moreover, deletion of the CaSR from keratinocytes reduces their stores of calcium and like the deletion of VDR blocks their response to extracellular calcium (Cao) including the formation of the Cdh1/Ctnn complex [60, 61]. In addition to interfering with PKCα activation and Cdh1/Ctnn complex formation, this decrease in Cai is likely to interfere with the operation of the store operated calcium channel (SOC). The SOC is comprised of the membrane bound Orai1 that as a tetramer forms the pore forming unit in the plasma membrane. This pore is activated by the ER calcium sensor stromal interacting molecule 1 (Stim1). When the ER levels of calcium are depleted, Stim1 interacts with Orai1 in the membrane to stimulate calcium entry [62, 63]. Injuring the keratinocyte is expected to be one such stimulus for ER calcium depletion and SOC activation. If SOC function is blocked by inhibitors or deletion of Orai1, keratinocyte proliferation and migration are impacted [64].

## Wnt/Ctnnb signaling

The Wnt/Ctnnb (b-catenin) signaling pathway likewise plays a major role in the wounding response as recently reviewed [65]. This pathway is activated by wounding, and contributes to the proliferative response of both fibroblasts and keratinocytes, their migration, and the re-epithelialization of the wound. In general, the ability of Ctnnb to promote proliferation involves its genomic actions in association with its nuclear partners LEF/TCF, whereas its actions in migration and re-epithelialization are as part of the Cdh1/Ctnn complex in the membrane. The interaction between Ctnnb and VDR is complex and appears to differ between events within the SC of the HF and the IFE. In colon cancer cells the VDR has been shown to bind to Ctnnb and reduce its transcriptional activity in a ligand dependent fashion [66]. Furthermore, in these cells 1,25(OH)_2_D_3_ has been shown to increase Cdh1 expression, such that Ctnnb is redistributed from the nucleus to the plasma membrane where it forms a complex with Cdh1 and other catenins at adherens junctions[67]. However, the suppression of Ctnnb signaling by 1,25(OH)_2_D_3_ does not necessarily require Cdh1 [68]. Rather Ctnnb binds to VDR in its AF-2 domain, binding that enhances the ability of 1,25(OH)_2_D_3_ to activate the transcriptional activity of the VDR [68] but blocks the transcriptional activity of Ctnnb. Similar processes are likely to be occurring in keratinocytes. Palmer et al. [69] evaluated the interaction between VDR and Ctnnb in transcriptional regulation, and identified putative response elements for VDR and β-catenin/LEF in a number of genes including *Shh*, *Ptch1* and *2, Gli1* and *2.* In epidermal keratinocytes knockdown of the VDR reduces Cdh1 expression and formation of the Cdh1/Ctnn membrane complex resulting in increased Ctnnb transcriptional activity. 1,25(OH)_2_D_3_, on the other hand, inhibits Ctnnb transcriptional activity in epidermal keratinocytes in part by stimulation of Cdh1 transcription and membrane localization. However, other studies suggest that VDR may potentiate, not inhibit Ctnnb transcriptional activity in the hair follicle [31]. Cianferotti et al. [31] found a reduction in proliferation of keratinocytes in the dermal portion of the hair follicle (below the bulge) in VDR null mice, and no stimulation of proliferation when Ctnnb was overexpressed in these cells in contrast to the stimulation of proliferation in control animals. These authors conclude that the VDR promotes Ctnnb function at least in the hair follicle. Differences in the actions of Ctnnb in the epidermis and hair follicle were originally demonstrated in studies from the Fuchs’ laboratory [70], and are consistent with the possibility that 1,25(OH)_2_D_3_ and VDR exert different effects on Ctnnb signaling in the hair follicle and epidermis. How these differences impact vitamin D regulated wound healing, and in particular the responses of the SC in the different niches remains unclear.

## Role of coactivators and other transcription factors

The actions of VDR in the skin and in wound healing are regulated by a variety of transcription factors and co-regulators. The Mediator complex plays a major role in regulating VDR actions in keratinocyte proliferation and early differentiation, whereas the steroid receptor co-regulator complexes have a greater effect on the later stages of keratinocyte differentiation [10]. The interactions of these co-regulators are not specific for VDR, however, so that studies involving their deletion or overexpression are not specific for their effects on VDR function. That said deletion of *Med 1*, that codes for the unit of the Mediator complex that binds directly to VDR, actually promotes keratinocyte proliferation and accelerates re-epithelialization [71], due in part to suppressing TGFb expression and function [72]. In combination the deletion of both *Med 1* and *Vdr* reverses the inhibition by *Vdr* deletion on keratinocyte proliferation and formation of the permeability barrier [73]. The role of the steroid receptor complexes on skin wound healing has received less attention, although deletion of *Src 3* was found to delay wound healing [74]. RXR, the major partner of VDR in most of its genomic functions in the skin and elsewhere, has likewise received minimal study with respect to its role in wound healing.

On the other hand p63 is likely to play a major role in VDR regulation of the wounding response. P63 induces VDR by direct binding to the *VDR* promoter [75]. Like *VDR*, deletion of *p63* blocks epidermal differentiation [76]. Kouwenhoven et al. [77] used RNA-seq and RNAPII ChIP seq to determine which of the thousands of p63 binding sites were associated with ongoing transcription during keratinocyte differentiation. Not surprisingly most were not, but of those associated with active gene transcription the authors identified potential partnering transcription factors of which VDR was one, and these sites were enriched in super enhancers considered important for SC fate determination.

P63 has two main classes of isoforms as a result of alternate promoters. Transcriptionally active (TA) p63 includes all 14 exons. The ∆Np63 has its promoter in an intron downstream of exon 3 which though it lacks the TA domain (exons 1–3) it retains the ability to bind to DNA and induce genes [78]. TAp63 and ∆Np63 bind to the same DNA element. It is the ∆Np63 isoform that is most highly expressed in the basal layer of the epidermis and is critical for keratinocyte differentiation [79]. ∆Np63 is also expressed in the outer root sheet, bulge, and hair matrix of the HF [80]. In the scRNA seq study of wounding by Haensel et al. described earlier ∆Np63 along with Col17a mark the basal SC cluster. Knockdown of ∆Np63 in the basal layer of the epidermis impairs wound healing [80].

## Summary

The skin provides the major barrier to the life threatening forces in the environment, keeping in what we need kept in and keeping out that which is harmful. Disrupting this barrier leads to loss of bodily fluids—water, electrolytes, blood—and provides a portal for infectious organisms. Therefore, it is essential that wound healing be efficient and at the same time protect the body from both loss of essential fluids and infection during the healing process. The normal skin does this quite well. Among the important regulators of these functions in skin are vitamin D and calcium. Vitamin D and calcium facilitate the formation of the permeability barrier, protecting against losses of fluids, and promoting the innate immune response, thus protecting against infection. Vitamin D deficiency is observed in many patients with poorly healing wounds, and dressings that combine both calcium and vitamin D show benefit indicating that further understanding of how vitamin D and calcium signaling promote wound healing will have a major clinical impact on this population.

Within the epidermis and HF are located niches of SC which under steady state conditions are responsible for the maintenance of the epidermis, sebaceous glands and HF cycling, respectively. Upon wounding the fate of these SC changes, directing them toward restoration of the epidermis. The molecular events that occur to enable this change in cell fate are not all that well understood. However, these changes appear to involve both rapid changes in calcium and Wnt/Ctnnb signaling and genomic changes within these cells redirecting their fate enabling their migration to the wound site to re-epithelialize the wound with subsequent regeneration of the epidermis. VDR is expressed in these SCs, and deletion of *VDR* from these cells retards their ability to respond to wounding. The function of VDR is regulated by a number of co-regulators, the best studied in skin are the Mediator and steroid receptor co-activator complexes. A number of genes implicated in cell fate determination are regulated by super enhancers, which are enriched in transcription factors such as p63, which like VDR when deleted retards the response to wounding. P63 like VDR is expressed in these SCs, and for a number of cell fate genes VDR and p63 colocalize in their promoters. Our premise is that coordination between calcium, Ctnnb, VDR, and p63 regulate the shift in cell fate of the epidermal and HF SCs to enable the re-epithelialization of the wound and the subsequent regeneration of the epidermis.
